# Coexistence of cicatricial alopecia and alopecia areata: A case series

**DOI:** 10.1016/j.jdcr.2026.04.061

**Published:** 2026-05-06

**Authors:** Meshi Paz, Caroline Kreytak, Aubrey Martin, Raymond Z. Ezzat, Divya Sharma, Artur Zembowicz, Jamie MacKelfresh, Maryanne M. Senna

**Affiliations:** a Division of Dermatology, Lahey Hospital and Medical Center, Burlington, Massachusetts; b Department of Dermatology, Emory University School of Medicine, Atlanta, Georgia; c Department of Dermatology, Harvard Medical School, Boston, Massachusetts

**Keywords:** alopecia areata, cicatricial alopecia, fibrosing alopecia, fibrosing alopecia in a pattern distribution, lichen planopilaris

Cicatricial alopecia (CA) and alopecia areata (AA) are distinct autoimmune conditions that target different regions of the hair follicle: the bulge and bulb, respectively. Although both are well-characterized individually, their coexistence is rarely reported. Prior cases describe single instances of AA arising with frontal fibrosing alopecia and AA occurring alongside CA.^1^, ^2^, ^3^ Here, we present the largest series to date of 5 biopsy-confirmed patients with concurrent AA and CA, encompassing multiple types of CA and noting disease course and treatment outcomes, including Janus kinase (JAK) inhibition. By expanding on the existing knowledge, we hope to offer insight into clinical overlap and potential shared disease pathomechanism.

Patients were seen at Lahey Hospital and Medical Center (*n* = 3) and Emory University Hospital (*n* = 2). Ages ranged from 28 to 76 years. All had biopsy-confirmed scarring alopecia: frontal fibrosing alopecia, lichen planopilaris, or fibrosing alopecia in a pattern distribution. All patients also had clinical and/or histologic evidence of AA, including alopecia totalis, ophiasis, and patchy involvement (Table I).

**Table I:** Summary of clinical characteristics of patients with concurrent alopecia areata and cicatricial alopecia

Characteristic	Patient 1∗	Patient 2	Patient 3	Patient 4	Patient 5
Sex	Female	Female	Female	Male	Female
Age (y)	65	68	76	28	66
Cicatricial alopecia type	FFA	LPP	FAPD	LPP	FFA
Year of CA diagnosis	2024	2019	2020	2019	2020
AA pattern	Alopecia totalis	Ophiasis AA	Ophiasis AA	Alopecia universalis	Patchy AA
Year of AA diagnosis	2022	2019	2020	2004	2024
Immune history	Eczema, anemia, hypothyroidism	Asthma, allergies, endometriosis	Severe ITP	Celiac disease, asthma	None
Diagnosis progression	AA → CA	Concurrent diagnosis	Concurrent diagnosis	AA → CA	CA → AA
Family history of AA	None	Father with alopecia totalis	Mother and other relatives with AA	Not recalled by patient	Yes (unspecified)
Site of overlap	Frontal scalp	Vertex	Vertex	Vertex	Frontal scalp
Trichoscopic/histologic findings	Biopsy with interface CA consistent with LPP/FFA, perifollicular erythema, and scale	Decrease in sebaceous glands, lymphocytes in a perifollicular location at the level of the isthmus, perifollicular fibrosis with rare dyskeratotic follicular epithelial cells, and miniaturized follicles	Biopsy consistent with scarring alopecia, broken hairs, and yellow dots	Perifollicular scale, pustules, erythema, and evidence of follicular dropout	Biopsy-confirmed FFA and smooth patches with no peripilar scale, suggestive of AA
Duration of AA (mo)	≥25	60	60	252	9
AA flare status at follow-up	Resolved	Flare (2024)	Resolved	Resolved	Resolved
Treatment response	Full regrowth on baricitinib 4 mg; stable on topical mometasone 0.1% solution	Stable on ILK and topical clobetasol solution	Stable on baricitinib and oral minoxidil	Stable on ILK, oral minoxidil, and topical clobetasol solution	Stable on ILK

*AA*, Alopecia areata; *CA*, cicatricial alopecia; *FAPD*, fibrosing alopecia in a pattern distribution; *FFA*, frontal fibrosing alopecia; *ILK*, intralesional triamcinolone; *ITP*, immune thrombocytopenic purpura; *LPP*, lichen planopilaris.

∗ Histopathology of biopsy samples from patient 1 is shown in Figure 1.

One patient with alopecia totalis experienced complete regrowth with baricitinib 4 mg daily for 9 months. After self-discontinuation, she experienced perifollicular erythema and scale on the frontal scalp, with biopsy-confirmed frontal fibrosing alopecia. Another patient with long-standing alopecia universalis experienced new vertex inflammation consistent with lichen planopilaris while receiving treatment for AA. In 2 patients, scalp biopsies from areas previously affected by AA demonstrated superimposed scarring alopecia. Autoimmune comorbidities (eg, asthma, hypothyroidism, and celiac disease) were present in all patients. Three had first-degree relatives with AA.

These findings support the clinical and immunologic intersection between AA and scarring alopecias, despite the small sample size. Although AA typically targets the bulb and CAs target the follicular bulge, both involve the immune privilege collapse and T-cell–mediated inflammation.^4^ Shared immune pathways, such as the Janus kinase-signal transducer and activator of transcription axis and interferon gamma signaling, may contribute to these overlapping presentations.^4^ In several patients, scarring alopecia arose after or at AA sites, suggesting a possible progression from nonscarring to scarring disease through immune reshaping, chronic inflammation, or treatment-related immune shifts.

Dermatoscopic and histopathologic evaluations were essential to recognizing overlapping diseases. One patient (*n* = 1) had biopsy-confirmed CA and AA from different scalp regions, whereas the remaining (*n* = 4) had biopsy-confirmed CA with strong clinical presentations of AA, including ophiasis pattern (*n* = 2), long-standing alopecia universalis preceding CA (*n* = 1; Fig 1, *A* and *B*), and delayed-onset patchy AA without clinical inflammation (*n* = 1). Although JAK inhibition produced complete regrowth in 1 severe AA case, the scarring component developed after discontinuation of JAK inhibitor therapy and was managed by topical corticosteroids and intralesional triamcinolone. The response to JAK inhibition, therefore, appeared limited to AA.

**Fig 1:**
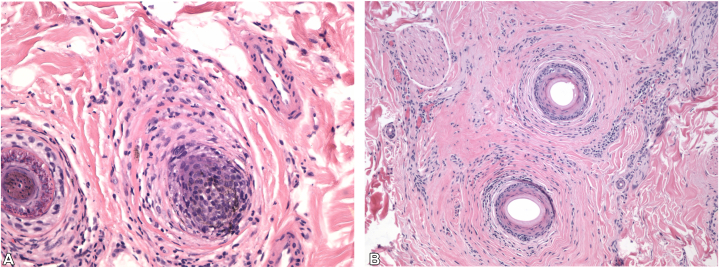
Histopathologic correlation of biopsies from patient 1. **A,** Left crown scalp demonstrating peribulbar lymphocytic infiltrate, consistent with AA. **B,** Right frontal hairline demonstrating perifollicular lymphocytic inflammation and fibrosis. Consistent with scarring alopecia, specifically frontal fibrosing alopecia.

To our knowledge, this is the largest series to date of concurrent AA and CA. Clinicians should maintain a high index of suspicion for dual pathology in patients with evolving or refractory hair loss. These findings highlight the importance of scalp biopsy to guide diagnosis and suggest that shared or sequential immune pathways may target multiple hair follicle compartments in some patients with alopecia.

## Conflicts of interest

None disclosed.
